# Correlation Between Meteorological Factors and the Incidence of Emergency Operations: A Retrospective Observational Study at 24 Hospitals in Japan

**DOI:** 10.7759/cureus.87597

**Published:** 2025-07-09

**Authors:** Masaki Orihara, Tomonori Takazawa, Takashi Suto, Chizu Aso, Shigeru Saito

**Affiliations:** 1 Department of Anesthesiology, Gunma University Graduate School of Medicine, Maebashi, JPN; 2 Department of Anesthesiology, Faculty of Medicine, University of Toyama, Toyama, JPN

**Keywords:** atmospheric pressure, emergency operation, humidity, japan, temperature

## Abstract

Background

Although meteorological changes can affect the incidence of the onset of certain diseases, no study has investigated the correlation between meteorological factors and emergency operations. This study aimed to examine a possible association between meteorological factors and the need for anesthetic care for emergency surgery. We hypothesized that changes in atmospheric pressure, temperature, and humidity would be associated with the daily volume of emergency surgeries.

Methods

This retrospective multicenter observational study of emergency operations performed between 2009 and 2017 at 24 Japanese hospitals included a total of 2,423 emergency operations. Meteorological data, including daily mean values of atmospheric pressure, temperature, and humidity, were obtained from the Japan Meteorological Agency. Statistical analysis was performed using univariate and multivariate ordinal logistic regression to elucidate the correlation between the incidence of emergency operations and meteorological factors.

Results

The most common types of operations were gastrointestinal surgery (n = 1161; 47.9%), followed by neurosurgery (n = 496; 20.5%), and obstetrics and gynecology procedures (n = 419; 17.3%). Multivariate ordinal logistic regression analysis revealed a correlation between daily mean atmospheric pressure (OR: 1.0131, 95% CI: 1.0009-1.0255, P = 0.0352) and the number of emergency operations, with the number of emergency operations increasing as daily mean atmospheric pressure increased. Further, multivariate ordinal logistic analysis showed that an increase in mean humidity compared to the previous day was associated with an increase in the number of emergency operations for subarachnoid hemorrhage (OR: 1.1944, 95% CI: 1.0228-1.3966, P = 0.0252).

Conclusion

These results suggest that meteorological factors might enable anesthesiologists to predict the kinds of emergency surgeries likely to be scheduled. However, the mechanisms underlying these findings need to be clarified.

## Introduction

Organisms, including humans, possess homeostatic regulatory systems to maintain good health and protect themselves from external environmental changes, although the mechanisms are not always completely effective. Indeed, weather is believed to influence various biological events [1]. Diseases that occur as a result of weather changes influencing the human body are called meteorological diseases [2]. There is considerable evidence showing that meteorological factors, such as atmospheric pressure, temperature, and humidity, affect the incidence of various diseases. For example, lower temperatures are associated with a higher risk of hemorrhagic stroke [3-5], likely because exposure to cold increases peripheral vasoconstriction and blood pressure [6,7].

Some diseases often require emergency surgery. However, it is challenging for anesthesiologists to predict when an emergency operation requiring anesthesia is likely to occur. The outcomes of some conditions are affected by how quickly surgery is performed following their onset [8,9]. In addition, limited availability of resources, especially outside routine work hours, might also affect outcomes. Therefore, the ability to predict the need for emergency operations could benefit hospital resource allocation and staffing. The use of meteorological data might contribute, to some extent, to predicting the need for surgery in conditions affected by environmental factors [10]. However, to the best of our knowledge, no previous studies have investigated the correlation between meteorological factors and the occurrence of emergency operations. This study, therefore, aimed to examine a possible association between meteorological factors and the need for anesthetic care in emergency surgery. We hypothesized that changes in atmospheric pressure, temperature, and humidity would be associated with the volume of emergency surgeries requiring anesthetic management.

## Materials and methods

Emergency operations

This retrospective observational study was approved by the ethics committee of Gunma University Hospital (ID: HS2017-239). The study was registered with the University Hospital Medical Information Network Clinical Trials Registry (ID: UMIN000032582). This article adheres to the Strengthening the Reporting of Observational Studies in Epidemiology (STROBE) guidelines. The Department of Anesthesiology at Gunma University Hospital has approximately 40 anesthesiologists and is responsible for dispatching anesthesiologists to 24 nearby hospitals for emergency operations on weeknights and holidays, as needed. All hospitals are located within 50 km of Gunma University Hospital. The decision for emergency surgery was made by the attending physicians or surgeons in each department, based on clinical judgment and institutional protocols. Data on emergency operations performed from April 1, 2009, to March 31, 2017, were collected from these hospitals, based on records maintained by liaising physicians. We began accessing the data on May 15, 2018. The collected data included the date of operation, diagnosis, operative procedure, and the name of the hospital where the operation was performed. We studied cases in which emergency surgery was performed by the departments of gastrointestinal surgery, neurosurgery, obstetrics and gynecology, orthopedic surgery, urology, and cardiovascular surgery.

Meteorological data

Meteorological data from April 1, 2009, to March 31, 2017, were obtained from the Japan Meteorological Agency. We examined meteorological data published online, including atmospheric pressure, temperature, and humidity recorded by the Maebashi Local Meteorological Observatory, which is located in front of Gunma University Hospital. We computed the daily averages of hourly meteorological data and used them in our analyses.

Annually, the four seasons were defined as follows: spring, March through May; summer, June through August; autumn, September through November; and winter, December through February.

Statistical analysis

Statistical analysis was performed using univariate and multivariate ordinal logistic regression to elucidate the correlation between the incidence of emergency operations and meteorological factors. The objective variable was the number of emergency operations per day, rather than simply the presence or absence of surgery. Because this variable represents ordered categories with equal intervals, ordinal logistic regression was selected as an appropriate analytical method to preserve the ordinal nature of the outcome. Since we considered that weather changes might affect the number of emergency surgeries, we analyzed changes in mean air pressure, temperature, and humidity relative to the previous day, in addition to their absolute values. The primary analysis focused on the total number of emergency surgeries. In addition, we conducted exploratory subgroup analyses focusing on specific surgical categories. In these analyses, the objective variables were the number of emergency operations in individual departments and emergency operations by disease. Statistical analysis of seasonal variations in emergency operations was performed using the Kruskal-Wallis test.

Statistical tests were considered significant at P < 0.05, and all P values were two-sided. Data were analyzed using JMP 11 software (SAS Institute Inc., Cary, North Carolina) and R (The R Foundation for Statistical Computing, Vienna, Austria).

## Results

Overview of emergency operations

A total of 2,423 emergency operations were assessed in this study, including 1,161 (47.9%) gastrointestinal surgeries, 496 (20.5%) neurosurgeries, 419 (17.3%) obstetric and gynecologic procedures, 197 (8.1%) orthopedic surgeries, 53 (2.2%) urologic surgeries, and 40 (1.7%) cardiovascular surgeries (Table 1). No cases were excluded due to ineligibility, refusal to participate, or other reasons.

**Table 1:** Details of emergency operations performed during the observation period

Department	Diagnosis	Number of cases	Incidence (in department)
Gastrointestinal surgery	Appendicitis	374	15.4 (32.2)
	Perforation of the digestive tract	261	10.8 (22.5)
	Ileus	204	8.4 (17.6)
	Hernia incarceration	158	6.5 (13.6)
	Others	164	6.8 (14.1)
	Subtotal	1161	47.9 (100.0)
Neurosurgery	Subarachnoid hemorrhage	242	10.0 (48.8)
	Intracerebral hemorrhage	201	8.3 (40.5)
	Subdural and epidural hematoma	12	0.5 (2.4)
	Brain infarction	10	0.4 (2.0)
	Others	31	1.3 (6.3)
	Subtotal	496	20.5 (100.0)
Obstetrics and gynecology	Cesarean section	368	15.2 (87.8)
	Ectopic pregnancy	25	1.0 (6.0)
	Torsion of ovarian pedicle	16	0.7 (3.8)
	Others	10	0.4 (2.4)
	Subtotal	419	17.3 (100.0)
Orthopedic surgery	Bone fracture	81	3.3 (41.1)
	Dislocation	43	1.8 (21.8)
	Others	73	3.0 (37.1)
	Subtotal	197	8.1 (100.0)
Urology	Testicular torsion	24	1.0 (45.3)
	Perineal trauma	12	0.5 (22.6)
	Others	17	0.7 (32.1)
	Subtotal	53	2.2 (100.0)
Cardiovascular surgery	Aortic dissection	14	0.6 (35.0)
	Ruptured aortic aneurysm	11	0.5 (27.5)
	Others	15	0.6 (37.5)
	Subtotal	40	1.7 (100.0)
Others		57	2.3 (100.0)
Total		2,423	100.0

**Figure 1:**
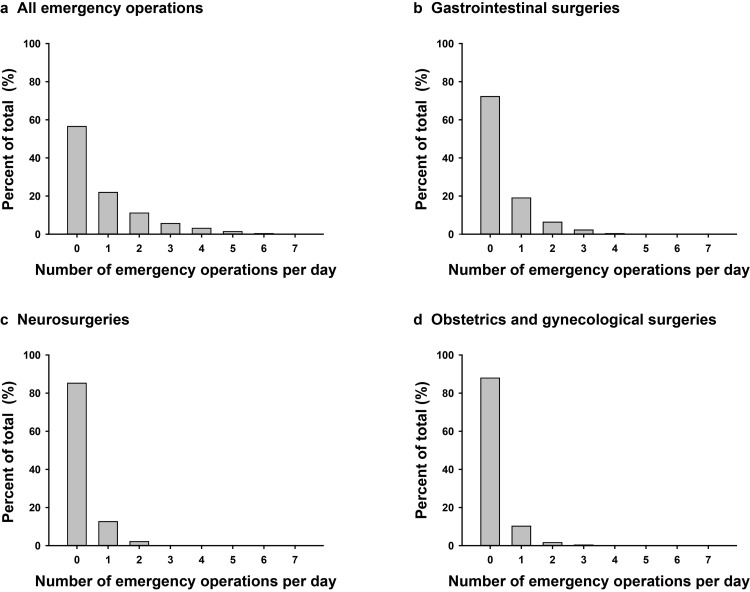
Distributions of the number of emergency operations per day

The reasons for emergency operations performed by the gastrointestinal surgery department were appendicitis, perforation of the digestive tract, ileus, and hernia incarceration, in that order. The most common reason for emergency operations performed by the neurosurgery department was subarachnoid hemorrhage (SAH), followed by intracerebral hemorrhage. The most common emergency operations in the department of obstetrics and gynecology were cesarean sections. Details of the emergency operations performed, including those by departments other than the three mentioned above, are shown in Table 1. The number of all emergency operations per day varied widely, from zero to seven. Days on which no emergency surgeries were performed were the most common. The number of gastrointestinal surgeries, neurosurgeries, and obstetric and gynecologic operations per day ranged from zero to five, zero to four, and zero to three, respectively. In all departments, days without emergency surgery were most frequent (Figure 1).

Observed meteorological data

**Figure 2:**
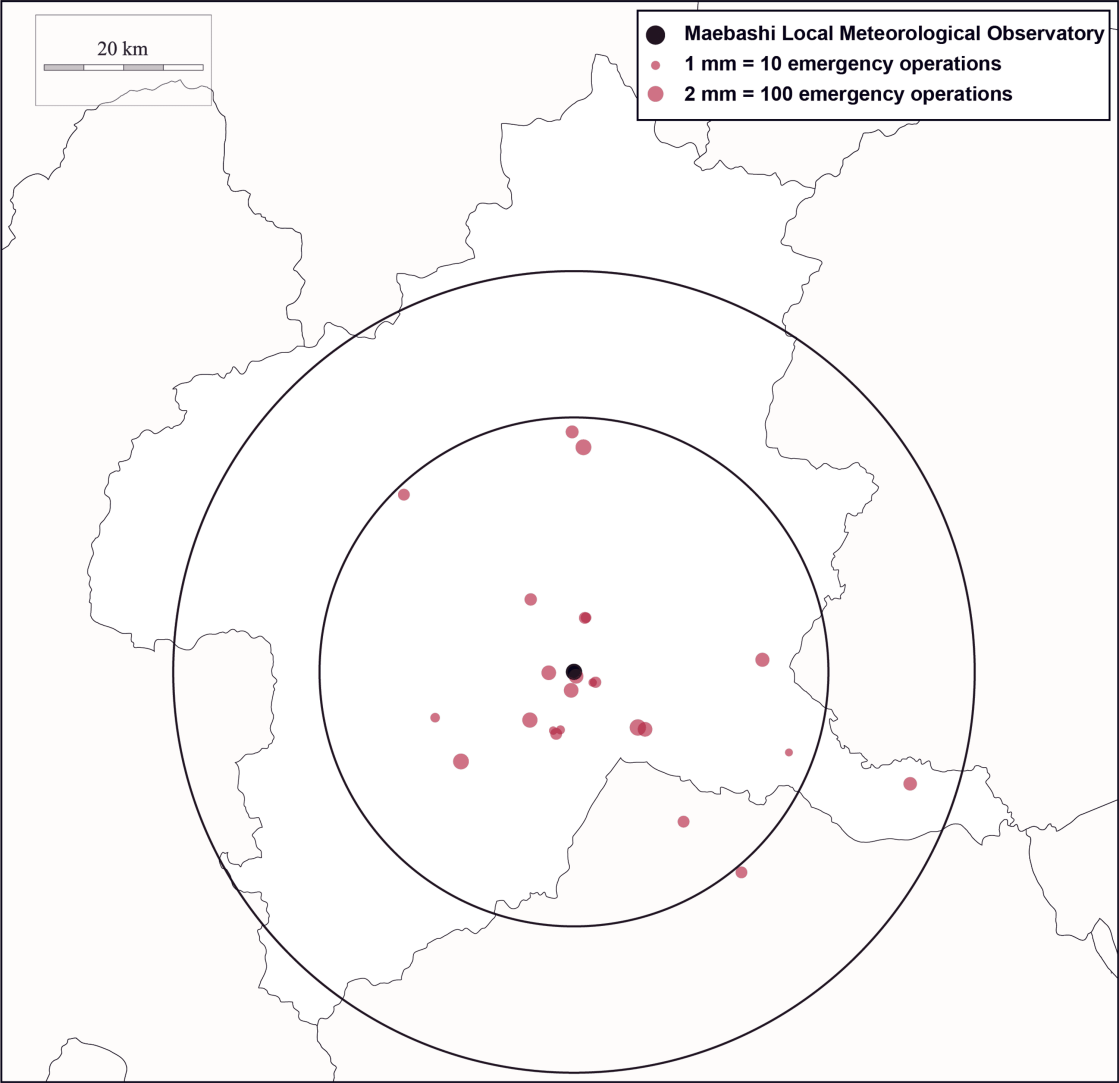
Location of the Maebashi Local Meteorological Observatory and 24 hospitals near Gunma University Hospital The two black circles represent distances of 30 km and 50 km centered at the Maebashi Local Meteorological Observatory. The red dots indicate the locations of the 24 participant hospitals, with the size of each red dot indicating the total number of emergency operations performed at each hospital. Map of Gunma Prefecture by Chizu-Seisaku.com, used under CC BY 4.0.

Maebashi city is located roughly in the center of the main Japanese islands and has a humid subtropical climate (Figure 2).

**Figure 3:**
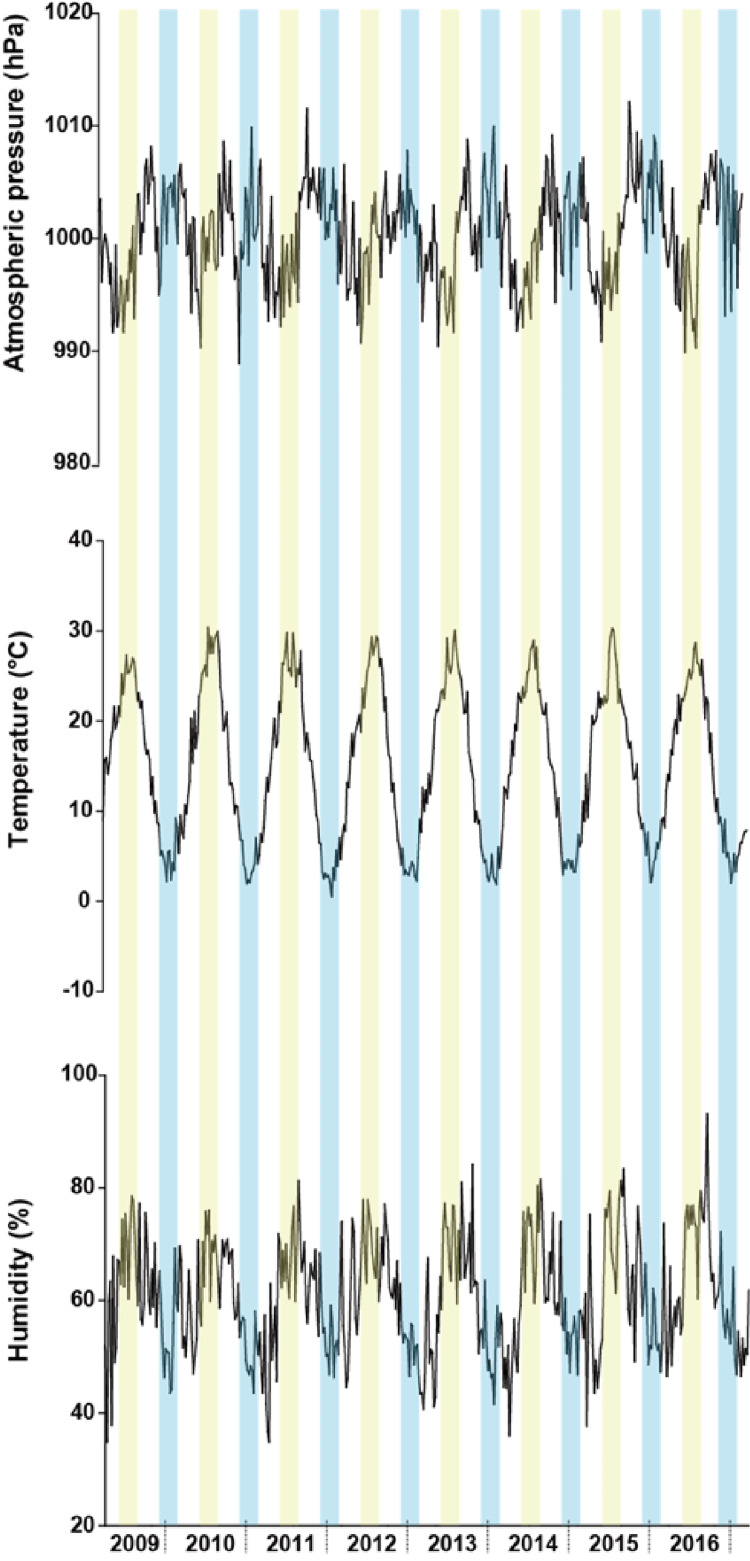
Average changes in atmospheric pressure, temperature, and humidity over the study period The seasons in the graph follow in the order of spring, summer, autumn, and winter. The yellow zones represent summer, and the blue zones represent winter. The white zones to the left and right of the yellow zones indicate spring and autumn, respectively.

The observed mean atmospheric pressure was 1000.4 ± 6.7 hPa (mean ± SD), and humidity was 60.7 ± 14.3% over the entire study period. The observed mean temperature was 15.2 ± 8.7 °C for the entire period, ranging from an absolute minimum of −1.9 °C on January 15, 2017, to an absolute maximum of 32.6 °C on August 10, 2013. Over the eight-year study period, the average changes in atmospheric pressure, temperature, and humidity within each season were similar (Figure 3).

Summer tended to have low atmospheric pressures, high temperatures, and high humidity. Winter tended to have high atmospheric pressures, low temperatures, and low humidity.

Correlation between meteorological factors and the incidence of overall emergency operations

Univariate ordinal logistic regression showed no significant differences between the number of emergency operations and meteorological factors. On the other hand, multivariate ordinal logistic regression analysis revealed an association between daily mean atmospheric pressure (OR: 1.0131, 95% CI: 1.0009-1.0255, P = 0.0352) and the number of emergency operations, which increased as the daily mean atmospheric pressure increased. The details of these results are shown in Table 2.

**Table 2:** Results of univariate and multivariate ordinal logistic regression analysis of emergency operations OR, odds ratio; CI, confidence interval.

Explanatory variables	Univariate logistic regression			Multivariate logistic regression		
	OR	95% CI	P	OR	95% CI	P
Daily mean atmospheric pressure	1.0103	0.9997-1.0210	0.0561	1.0131	1.0009-1.0255	0.0352
Daily mean temperature	1.0019	0.9939-1.0100	0.6351	1.0051	0.9957-1.0146	0.2835
Daily mean humidity	1.0004	0.9956-1.0053	0.8436	1.0012	0.9953-1.0071	0.6846
Increase in mean atmospheric pressure compared to the previous day	1.0385	0.9683-1.1138	0.2898	1.0117	0.9351-1.0946	0.7711
Increase in mean temperature compared to the previous day	0.9827	0.9163-1.0539	0.6249	0.9775	0.9054-1.0555	0.5613
Increase in mean humidity compared to the previous day	0.9926	0.9256-1.0646	0.8368	0.9764	0.9025-1.0563	0.5519

Correlation between meteorological factors and the incidence of emergency operations in individual departments

In the analysis of emergency operations in individual departments, only those accounting for more than 10% of the total number of emergency operations, namely, gastrointestinal surgery, neurosurgery, and obstetrics and gynecology, were evaluated.

Univariate ordinal logistic regression analysis for neurosurgery revealed that the number of emergency operations was affected by the daily mean temperature (OR: 0.9875, 95% CI: 0.9759-0.9991, P = 0.0359) and by the increase in mean temperature compared to the previous day (OR: 0.8894, 95% CI: 0.8029-0.9851, P = 0.0247) (Table 3). These results can be interpreted as follows: the number of neurosurgery cases was lower when the average daily temperature was higher. Furthermore, an increase in the average temperature compared to that on the previous day was associated with a smaller number of neurosurgery cases. Univariate ordinal logistic regression analysis showed that the incidence of emergency surgeries performed by departments other than neurosurgery did not correlate with weather data (Table 3).

**Table 3:** Results of univariate ordinal logistic regression analysis of emergency operations in individual departments OR, odds ratio; CI, confidence interval.

Explanatory variables	Gastrointestinal surgery (n = 1161)			Neurosurgery (n = 496)			Obstetrics and gynecology (n = 419)		
	OR	95% CI	P	OR	95% CI	P	OR	95% CI	P
Daily mean atmospheric pressure	1.0056	0.9936-1.0178	0.3553	1.0048	0.9894-1.0203	0.5399	1.0018	0.9852-1.0186	0.8287
Daily mean temperature	1.0078	0.9986-1.0171	0.0957	0.9875	0.9759-0.9991	0.0359	0.9986	0.9859-1.0113	0.8301
Daily mean humidity	1.0033	0.9977-1.0089	0.2417	0.9997	0.9925-1.0068	0.9369	0.9950	0.9873-1.0027	0.2106
Increase in mean atmospheric pressure compared to the previous day	1.0328	0.9533-1.1190	0.4293	1.0492	0.9470-1.1624	0.3579	1.0053	0.8997-1.1234	0.9247
Increase in mean temperature compared to the previous day	1.0169	0.9386-1.1017	0.6816	0.8894	0.8029-0.9851	0.0247	0.9663	0.8649-1.0797	0.5455
Increase in mean humidity compared to the previous day	0.9883	0.9122-1.0706	0.7732	1.0880	0.9817-1.2057	0.1076	0.9696	0.8678-1.0834	0.5865

On multivariate ordinal logistic regression analysis, there were no departments for which the number of emergency operations was related to any of the meteorological factors (Table 4).

**Table 4:** Results of multivariate ordinal logistic regression analysis of emergency operations in individual departments OR, odds ratio; CI, confidence interval.

Explanatory variables	Gastrointestinal surgery (n = 1161)			Neurosurgery (n = 496)			Obstetrics and gynecology (n = 419)		
	OR	95% CI	P	OR	95% CI	P	OR	95% CI	P
Daily mean atmospheric pressure	1.0106	0.9966-1.0248	0.1366	0.9962	0.9787-1.0139	0.6728	1.0022	0.9832-1.0216	0.8214
Daily mean temperature	1.0085	0.9977-1.0195	0.1213	0.9876	0.9740-1.0013	0.0760	1.0035	0.9886-1.0185	0.6440
Daily mean humidity	1.0036	0.9969-1.0104	0.2825	1.0007	0.9921-1.0093	0.8656	0.9938	0.9845-1.0031	0.1961
Increase in mean atmospheric pressure compared to the previous day	1.0307	0.9420-1.1278	0.5095	1.0408	0.9271-1.1690	0.4988	0.9682	0.8543-1.0972	0.6130
Increase in mean temperature compared to the previous day	1.0161	0.9306-1.1095	0.7204	0.9230	0.8256-1.0319	0.1595	0.9403	0.8330-1.0615	0.3200
Increase in mean humidity compared to the previous day	0.9758	0.8917-1.0679	0.5953	1.0655	0.9492-1.1961	0.2816	0.9774	0.8630-1.1070	0.7198

Correlation between meteorological factors and the incidence of emergency operations classified by illness

Multivariate ordinal logistic analysis showed that an increase in mean humidity compared to the previous day was associated with an increase in the number of emergency operations for SAH (OR: 1.1944, 95% CI: 1.0228-1.3966, P = 0.0252) (Table 5).

**Table 5:** Results of multivariate ordinal logistic regression analysis of emergency operations performed in the neurosurgery department OR, odds ratio; CI, confidence interval.

Explanatory variables	Subarachnoid hemorrhage (n = 242)			Intracerebral hemorrhage (n = 201)		
	OR	95% CI	P	OR	95% CI	P
Daily mean atmospheric pressure	0.9898	0.9668-1.0135	0.3971	1.0063	0.9812-1.0323	0.6241
Daily mean temperature	0.9888	0.9709-1.0071	0.2328	0.9902	0.9706-1.0101	0.3326
Daily mean humidity	0.9958	0.9843-1.0073	0.4762	1.0074	0.9951-1.0199	0.2354
Increase in mean atmospheric pressure compared to the previous day	1.0448	0.8948-1.2209	0.5800	0.9892	0.8375-1.1691	0.8991
Increase in mean temperature compared to the previous day	0.9260	0.7975-1.0747	0.3123	0.9263	0.7880-1.0885	0.3529
Increase in mean humidity compared to the previous day	1.1944	1.0228-1.3966	0.0252	0.9620	0.8139-1.1367	0.6494

For cases other than SAH, the incidence of emergency surgeries by disease did not correlate with meteorological factors (data not shown).

Seasonal variations in emergency operations

**Figure 4:**
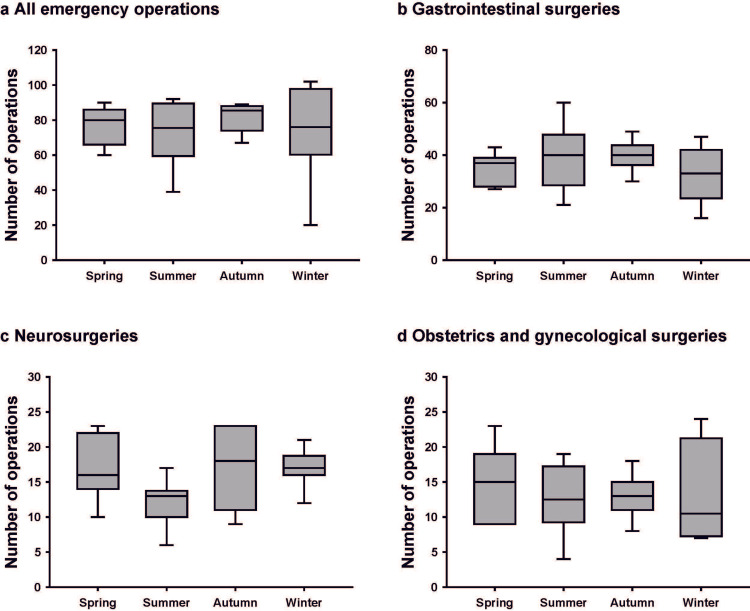
Seasonal variations in the number of emergency operations The ends of the boxes define the 25th and 75th percentiles, with the horizontal lines in the middle showing the mean, and the error bars defining the 10th and 90th percentiles.

Finally, we investigated the correlation between the season and the incidence of emergency operations. We observed no seasonal variation in the number of emergency operations overall or by department (Figure 4).

**Figure 5:**
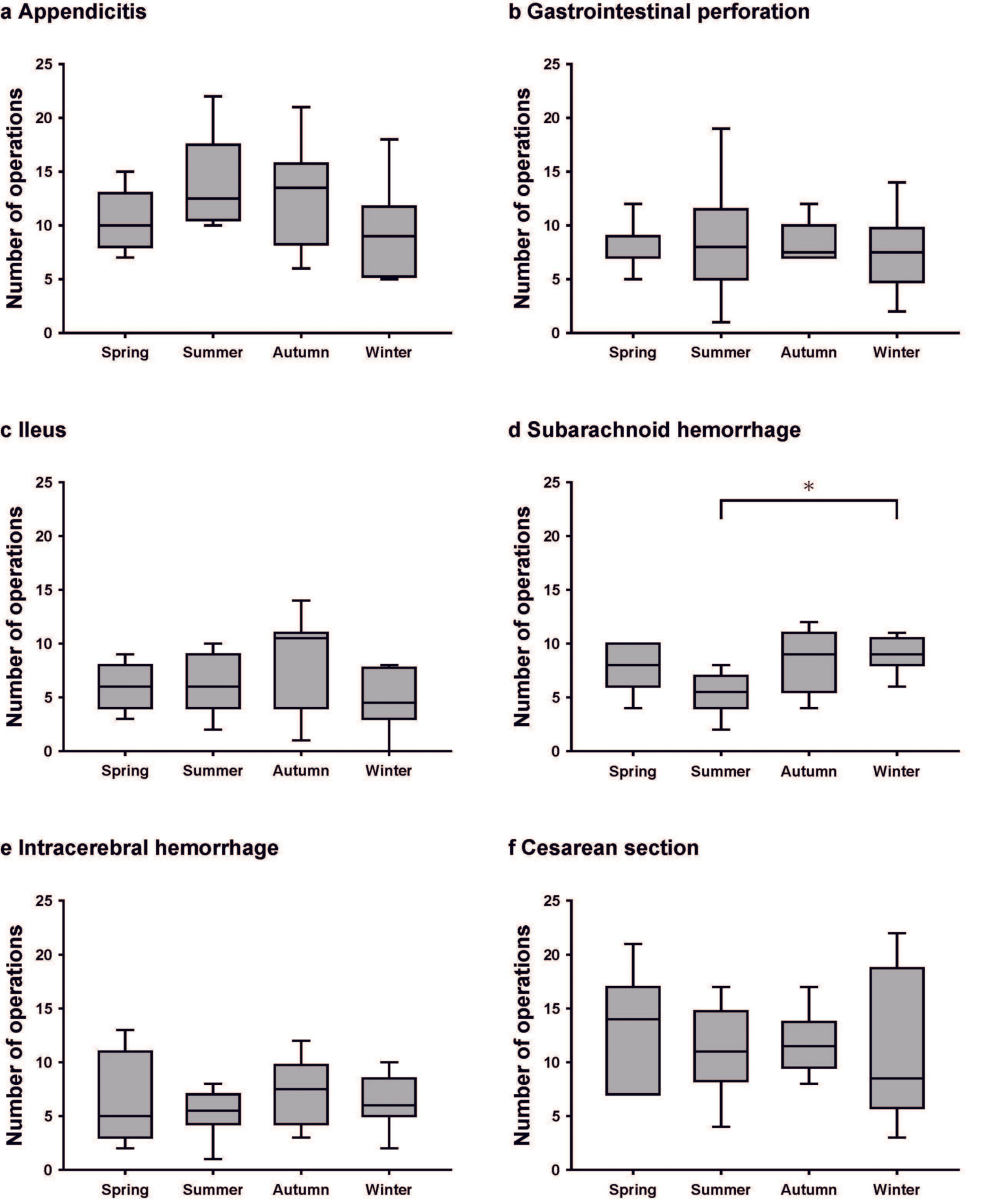
Seasonal variations in the number of emergency operations classified by illness The ends of the box define the 25th and 75th percentiles, with the horizontal lines in the middle showing the mean, and the error bars defining the 10th and 90th percentiles. *Kruskal-Wallis with post hoc Steel-Dwass test, P < 0.05.

Only the number of surgeries for SAH varied by season (Kruskal-Wallis test, P = 0.031), with the lowest number in summer and the highest number in winter (Figure 5). A significant difference was seen when comparing summer and winter (post hoc Steel-Dwass test, P = 0.021).

There were no seasonal differences in the number of operations for other diseases (data not shown).

## Discussion

This study explored the potential association between meteorological factors and the incidence of anesthetic care for emergency operations. Since meteorological parameters are interrelated, we discuss here those items that were significantly different in multivariate regression analysis. Although the observed associations were generally modest, our findings suggested that the overall number of emergency operations tended to increase as daily mean atmospheric pressure increased. Furthermore, a slight increase in the frequency of surgery for SAH was observed on days when humidity was higher than on the previous day. While these findings are statistically significant, their clinical implications remain limited. To the best of our knowledge, this is the first study to investigate the correlation between meteorological factors and emergency operations requiring the involvement of an anesthesiologist.

The mechanism by which higher atmospheric pressure increases the overall number of emergency operations is unclear from the current study. Notably, gastrointestinal surgery accounted for about half of the emergency operations evaluated. Since the composition of the emergency operations studied might have influenced the results, further research is needed to determine whether the findings of this study can be generalized to other countries and regions with different distributions of surgical cases.

Past studies have shown that weather affects emergency operations for specific diseases. We found that surgeries for SAH were significantly more common on days when humidity increased compared to the previous day (OR = 1.1944). A previous study conducted in Japan showed that mean humidity was associated with SAH occurrence [11]. Since SAH is life-threatening, the fact that emergency surgery is often performed on the day of SAH onset might have been a factor in the more significant association between meteorological data and surgery for SAH. SAH is a common condition among women in Japan, occurring more than twice as frequently in females as in males. A previous Japanese study reported that high average humidity was associated with the incidence of SAH in women, which is consistent with our findings. The authors of that study suggested a possible relationship between humidity and SAH, particularly in women, although the mechanism remains unclear [11]. The only observed seasonal difference in the number of surgeries in our study was for SAH, which was more common in winter than in summer (Figure 5). This finding is consistent with previous studies. The common perception regarding the mechanism for this correlation is that cold-induced hypertension may lead to SAH [11,12].

Another frequently occurring condition that requires discussion is appendicitis. It was the second most common cause of emergency surgery in this study, after cesarean section (Table 1). Acute appendicitis reportedly occurs more frequently during summer [13,14]. A study examining the relationship between appendicitis and temperature showed that appendicitis was more common in warmer temperatures [15]. Although our study did not show a significant difference, with a P-value of 0.0594, there was a weak correlation between high temperature and emergency operation due to appendicitis. Although the mechanism of the correlation between high temperatures and appendicitis is unclear, possible causes include changes in the content of the food consumed in summer and increased infections in the gastrointestinal tract due to higher temperatures [1]. The weak correlation between high temperature and emergency surgery due to appendicitis in the current study might be explained by the fact that appendicitis does not always require immediate operation. In fact, a recent study has shown that antibiotic treatment is noninferior to appendectomy [16].

We found no apparent correlation between the incidence of cesarean sections and meteorological factors. Environmental conditions might trigger uterine contractions through oxytocin release, and climatic conditions have been reported to influence labor [2,17,18]. Although there are many indications for cesarean section, we did not collect information on the reasons for performing cesarean section in this study. A future cesarean section analysis by cause might reveal a link to meteorological data.

Regarding the clinical implications of the results of this study, we believe that while the number of emergency operations is not affected by slight changes in weather conditions, it can change when weather conditions change significantly. For example, our results suggest that if the daily mean atmospheric pressure increases by 10 hPa, the number of emergency operations will likely increase 1.14-fold, and if the daily mean atmospheric pressure increases by 20 hPa, the number of emergency operations will increase 1.29-fold. Thus, if there are enough hospital resources and anesthesia staff, we recommend increasing the number of available staff on days with extreme weather conditions.

This study has several limitations. First, since meteorological data were collected at the Maebashi Local Meteorological Observatory for all hospitals, it might not accurately represent the conditions at each individual hospital. Although all 24 hospitals were located within approximately 50 km of this observatory, this may not fully capture local variations in weather conditions at each hospital. Therefore, while the data likely represent general meteorological trends in the study region, caution is warranted when interpreting these findings due to potential spatial heterogeneity. Second, there might be a variation in the time from consultation to the start of emergency operation between hospitals. Third, post-hoc power analysis for gastrointestinal surgeries and obstetric and gynecological surgeries, for which no significant relationship was found between meteorological factors and the number of emergency operations, revealed that none of the factors reached a power of 80%. In the future, evaluating a larger number of cases of these types of operations might indicate that meteorological factors do indeed affect the number of emergency operations. Fourth, this study included only cases that required emergency surgery, which may not reflect the full spectrum of disease incidence. Including nonsurgical cases in future research may provide a more comprehensive understanding. Potential confounding factors were not controlled for in this study. These unmeasured confounders may have influenced the observed associations and should be considered when interpreting the results. Further studies will be required to clarify the association between meteorological factors and the need for anesthetic care for emergency operations.

## Conclusions

This study suggests that meteorological factors might be helpful for anesthesiologists in predicting the likelihood of the need for anesthetic care in emergency operations. In addition, they might also be able to predict the kinds of diseases patients are likely to present with for emergency surgery, which would facilitate resource allocation. The mechanisms linking these events and meteorological factors need to be clarified.
